# New York State Medical Society

**Published:** 1853-04

**Authors:** 


### EDITORIAL DEPARTMENT.

*New York State Medical Society.* — The New York Medical Times, No. for March, 1853, contains a full account of the proceedings of the New York State Medical Society at the annual session in February last. The society remained in session for three days, and, in addition to topics of importance pertaining to the future usefulness of the organization, several subjects of scientific interest were introduced and discussed by the members present. We propose to notice the latter first, embracing the occasion to offer some remarks of our own thereupon. Afterward we will chronicle such other matters entering into the proceedings as will be likely to interest those of our readers residing in this state.

### *Croup.*

On this subject we quote as follows:

"Dr. Snyder, chairman of the committee, read a paper on Diphtheritic Croup—he gave the detail of thirty cases that had come under his observation—a disease that occurs in children between the ages of one and twelve years. It occurs more frequently in variable and damp weather, in the autumn and spring most often, never as an epidemic. This disease is not identical with the croup of adults. In the treatment of it, drastic cathartics and antimonials are usually injurious. Small and repeated doses of calomel; anodynes, to allay the irritant spasmodic action of the laryngeal muscles; the application of strong solution of nitrate of silver to the diseased part, and emetics of ipecac, are the best reliable means. In the success of tracheotomy he had no experience.

"Dr. Clark inquired the number of cases that recovered after the membrane had formed on the tonsils?

"Dr. Snyder said that in the fourteen who had died, the membrane had formed on the tonsils. None of these cases which recovered expectorated false membrane, though the other symptoms were similar to those where the membrane had formed. He thought the lymph was first exuded and formed on the palate or tonsils, and then extended downward upon the air passages.

"Dr. Clark gave an account of the microscopic structure of the exudation in cases which he had examined. The exudation is of a fibrous structure, and, he had observed, thicker at the upper part of the trachea in which it formed than at its lower part, where the formation was so thin and delicate that it could scarcely be seen by the naked eye. The examination he had made had been upon membranes expectorated, and also upon those found after death.

"Dr. Burwell thought the expectoration of the membrane in croup was exceedingly rare, and usually imperceptible.

"Dr. Clark said the membrane must be expectorated when it had formed, or the patient could not recover. This he considered as a truism.

"Dr. Shipman inquired for the experience of any person present, regarding the success of tracheotomy.

"Dr. Wood said the subject brought before the society by Dr. Snyder was one of great interest to the profession. With the profession it was looked upon as a fatal disease. Diphtheritic croup is different from spasmodic croup; and the child with the former may be playing with his fellow a few hours before, yet death in nine cases out of ten was then upon him. He had seen many cases of diphtheritic croup, but few of them recovered. He had seen none of them recover from the application of nitrate of silver. In New York, at the present time, it was treated with cinnabar fumigations, thus rapidly bringing the patient under slight mercurial influence. The great majority of operations (tracheotomy) were unsuccessful. In the majority of cases following scarlatina and erysipelas, it is œdema of the glottis which kills the patient, instead of the diptheritic croup.

"Dr. Guiteau said he thought it useless to perform the operation of tracheotomy at a late period in the course of the disease. It was almost always unsuccessful. He had resolved that in his next well-marked case he would perform it at an early period, and give the child almost the only chance of recovery.

"Dr. Wood said that where the membrane had commenced its formation on the tonsils, it would go down on the larynx into the bronchia and into the air cells, and the patient die. With regard to the time of the operation, there appeared to be but little difference; the result was almost alike successful.

"Dr. Snyder said he thought the only hope of success was in the early diagnosis—the discovery of the membranous formation before it had entered the larynx, and then arresting its farther progress. He did not mean to be understood that in his sixteen cases of recovery, this membrane had extended into the trachea and bronchia.

"Dr. Van Buren related a case which he has at present under treatment.

"Dr. A. W. Marsh said he had operated twice for membranous croup, both of which proved fatal, and he thought that in all cases where the inflammation had extended to the trachea, death is a natural consequence. The membrane is so rapidly formed that an operation does not afford permanent relief, and though the passage be opened, it is impossible to keep it so.

' Dr. Barker thought the operation more successful than had been reported. He hoped the observations of physicians would be continued through the year, and considered at the next annual meeting.

" Dr. Ayers related a case that came under his observation, in which the membrane was of an hour glass form, and the blood-vessels visible in it.

" Dr. Barker moved that the subject be laid on the table, to be brought up for discussion at the next annual meeting. Adopted."

The subject of croup is one of very great practical interest and importance. Perhaps there is no subject in practical medicine concerning which loose and incorrect pathological notions are so largely in vogue. We are surprised that in the remarks offered on the subject by the members present, no reference is made to the most valuable contributions by Dr. John Ware, of Boston. These contributions appear most unaccountably to be overlooked, to a great extent, by the medical profession of this country. An explanation of this fact may in part be derived from the small efforts that have been made by Dr. Ware and his friends to give his communications general circulation. They have been, however, neglected in quarters where this excuse cannot be considered to apply. The contributions to which we refer were published, some years ago, in this Journal, copied from the Boston Medical and Surgical Journal, (See No. for September, 1850.) The first and most valuable of several successful papers appeared originally in the New England Quarterly Journal of Medicine, more than ten years ago. In that paper Dr. Ware gave the results of an analysis of 131 cases of the several affections usually included under the name of croup, the records of which he had collected during twenty-five years of private practice. The results thus developed have established several most important inductions.

They established, *firstly*, that there are several pathological conditions with which are associated the croupy cough, viz: 1. Pure spasm of the laryngeal muscles; 2. Spasm accompanied by catarrh of the laryngeal mucous membrane; 3. Spasm with simple enythematic inflammation; and 4. A fibrinous exudation in the larynx, extending to the trachea, and frequently more or less over the bronchial tree.

They established, *secondly*, that the last named pathological condition is relatively infrequent in occurrence, the vast majority of cases of so-called croup involving one or more of the three conditions first mentioned.

They established, *thirdly*, that cases of croup involving pure spasm, or with catarrh, or with simple inflammation almost invariably end in recovery, and require very little in the way of treatment, while cases involving fibrinous exudation, (diphtheritic or true croup,) generally end fatally.

They established, *fourthly*, that exudative or true croup almost invariably is attended with a fibrinous exudation in the pharynx, while this exudation, in that situation, is absent in the several varieties of false croup; and, hence, the value of the presence or absence of this exudation in the pharynx as diagnostic criteria.

They established, *fifthly*, that cases of spasmodic, catarrhal, or simple inflammatory croup are not converted into true or exudative croup; but that whenever the latter form of disease exists, its distinctive character is intrinsic. Hence the error of the idea generally entertained, that all cases of croup, unless vigorously treated, will be likely to eventuate in the only serious or fatal form.

They established, *sixthly*, that the fibrinous exudation in *true* croup, occurs early in the disease, and already exists in the great proportion of cases when patients first come under treatment. Hence, the idea of preventing the formation of the false membrane is gratuitous.

They established, *seventhly*, that when cases of true croup end in recovery, the process by which a favorable issue obtains, is the separation of the false membrane by a puriform fluid produced beneath it, detaching it, and allowing its removal by expectoration; and that this process requires time — several days at least. Hence the notion that true croup can exist, and the false membrane dissolve in an imperceptible manner, or undergo absorption, is absurd. A case of croup in which no patches of false membrane are ever discoverable in the matter of expectoration, is therefore not a case of true croup.

In view of the practical bearing of the foregoing conclusions, it seems to us that Dr. Ware's contributions on this subject are entitled to be ranked among the most striking and valuable contained in the history of medicine for the past quarter of a century; and although there seems to be a reluctance, as yet, to attribute to them due importance, probably, in a great measure, because the profession, in general, are not acquainted with them, it may safely be predicted that Dr. Ware's name will, in all future time, be identified with the literature of this subject. For the correctness of the digest which we have thus hastily penned, we would respectfully refer our readers to the papers contained in the No. of this Journal already referred to.

In several communications made by Dr. Ware subsequent to the first paper published in 1842, he submits a plan of management of true croup different from that still pursued by the majority of practitioners. In this point of view the inductions presented deserve the most careful consideration. It would be altogether out of place to treat of this branch of the subject in the present connection. We have no intention of so doing, but we would commend it to the reflections of our readers; and as suggestives for thought we will briefly add a few considerations. The several varieties of croup which are devoid of any fatal tendency, there is reason to think, are frequently, if not generally treated by the vigorous employment of emetics, local and general bleeding, etc., either under the erroneous idea that they are cases of true croup, or that they will become so, unless the progress of the disease be arrested.

Dr. Ware may well ask "if this treatment does not do good, does it do no harm?" There can hardly be a doubt as to the proper answer to this question. We cannot resist the conviction that many children are thus sacrificed to the nimia diligentia. We beg practitioners who may read these remarks to ponder on this point; and in connection therewith we would take the liberty of recommending the little treatise by the late Prof. John B. Beck, entitled "Infant Therapeutics."

But suppose that we have clearly the responsibility of the management of a case of true or fibrinous croup, what are the ends to be kept in view in the treatment; in other words, what are the rational indications? Not to prevent the fibrinous exudation, for this has already taken place. Not to arrest inflammatory action, for this cannot be done here, more than in other situations. Not necessarily to abate the intensity of the inflammatory action, for it is not the intensity of the inflammation which places the patient in danger, but the product of inflammation — the fibrinous exudation. And here it may be remarked that the degree of inflammation is often, if not generally less in true croup, than in simple laryngitis. It is the *kind*, not the *degree* of inflammation which renders true croup so fatal.

The rational indications plainly relate to the prolongation of life till the separation and expectoration of the false membrane take place; to measures calculated to facilitate and hasten this process, and to the relief of urgent symptoms. Taking this view of the subject, Dr. Ware, and others have abandoned, in the treatment of croup, perturbatory and debilitating measures. These measures are as useless in *true* croup, as they are unnecessary in the several varieties of *false* croup. In both there is reason to think that they are destructive in their effects.

Rapid mercurialization, in order to secure the antiplastic effect attributed to this measure, fomentations, and the inhalation of watery vapor, are mainly relied upon, conjoined with anodynes to arrest, or hold in abeyance the spasmodic element which enters into the pathology of *true*, as of *false* croup, accounting for the paroxysmal occurrence of difficult respiration in the former, as well as the latter, and contributing not only to the suffering, but to the danger.

By reference to the late papers communicated by Dr. Ware, this new plan of treatment, pursued in a small number of cases, has been found more successful than the mode heretofore followed.

In this connection the subjects of tracheotomy, and the topical application of the nitrate of silver as proposed and practiced by Dr. Horace Green, are important. The latter measure, from several reported cases, seems to possess more efficacy than would appear from the observations of Dr. Wood referred to in the foregoing extract from the proceedings of the New York State Society. We have ourselves witnessed the apparent efficacy of this measure in a case which seemed to be desperate, occurring in the practice of Dr. Strong, of this city, and reported by him in a former No. of this Journal.

The views presented by Dr. Snyder as respects the treatment of croup, accord with those just presented; nor does it appear that exceptions were taken thereto by any of the members present.

Dr. S. is in error in stating that croup never occurs as an epidemic. Valleix gives instances of epidemic croup. We were recently informed by Prof. B. F. Palmer, of Woodstock, Vt., that an epidemic prevailed a few years ago, in a small village in his neighborhood, several cases occurring in rapid succession and proving fatal.

### *Puerperal Peritonitis.*

We find the following relating to the above subject:

"Dr. Van Dyck called upon Dr. Clark to continue his remarks, made to the Society last year, on Puerperal Peritonitis.

"Dr. Clark said: After the meeting of the Society, during the month of February, he had four cases in private practice. With these cases he had better success. They were seen by Drs. Rockwell, Smith, Gilman and Higgins. In none of these cases did he make the diagnosis first. Two or three drachms per hour of Magendie's solution of morphia, was the amount taken by some patients. Strange to say, when he got out of the hospital, such doses were not required. In no subsequent case was more than one grain or three-fourths of a grain of morphia required to keep the patient free from pain.

"Dr. Gilman has reported three cases treated in this way: one was unsuccessful. In the successful case he did not have the full charge of the patient.

"Dr. Williams, who has charge of the Lying-in Hospital, was unsuccessful, and thought he lost more patients by it than Dr. Clark had saved. The point is—the marked diminution of the quantity of opium to keep the patient narcotized until the pain and tenderness of the bowels subside, and the bowels are moved, and the pulse comes down from 160 to 140, or even from 180, as some of you have seen it, until any remaining symptom that would give special anxiety terminates. Cases occur in which the alarming symptoms continue ten or fifteen days, but because the narcotism was not carried to the point desired. The respiration in some cases is reduced to *seven per minute.* Unless the practice is brought to this point, it is not considered a fair trial. Whenever opium was used under my direction in the hospital, no pills that had been rolled more than six hours were administered, so that their hardness should not resist the action of the gastric juice. There is no particular choice between opium and morphia. He believed the disease did not prevail as it did last year; he had heard of none for several months. There had been a few cases in Williamsburgh. He commenced the treatment of the disease in its early stages with the doses he had named."

The method of treating peritonitis by the free exhibition of opium, first suggested, if we are correctly informed, by Dr. Armstrong, and practiced recently, with marked success, at the Bellevue Hospital, by Prof. Clark, is, in our view, one of the grand improvements in modern practical medicine. We do not express this opinion without due deliberation. We are cognizant of facts which, in our estimation, warrant such a conclusion. Observations bearing on this subject have not yet, to much extent, been submitted to the profession. We hope this will be done ere long. A full, authentic report of the cases treated by Dr. Clark is much to be desired. We hope he will soon favor the profession with such a report. We presume to remark that, after what has been said on the subject in various journals, this is due to himself, as well as to medical science.

It is a singular fact that cases in private practice did not require the large doses of opium administered to hospital patients. That all cases do not call for very large doses is shown by the case reported by the editor of this Journal in a former No.

We shall avail ourselves of another opportunity to enlarge on this subject.

### *Chloroform in Midwifery.*

"Dr. Burwell then read a paper on the use of chloroform in obstetrical practice. He had administered it in one hundred and eighty cases—one hundred and twenty-two of which were greatly relieved, fifty-five partially, and three got no relief from its use. In seventeen cases, labor was terminated by the use of the forceps,—in one case by craniotomy,—in one by turning. Eighty-eight of these cases were patients in labor for the first time. And in all these cases there had been not a single accident resulting from its use. He considered at length its effects upon the mental faculties and upon the muscular system—upon the pulse and upon the respiration. He then gave the general rules by which to be governed in its use, and particular directions about the manner in which it should be administered, and the quantity to be given at each dose.

"Prof. Barker confirmed the observations of Dr. Burwell in nearly every respect. He said that those patients who had full confidence in the good effect of chloroform rarely suffered any inconvenience from its use; while those who doubt its utility and are agitated before taking it, are not unfrequently affected with slight hysterical delirium, and require larger doses to bring them under its influence." * * * * *

"Dr. Spencer said he opposed the use of chloroform in obstetrical practice, more from theory than from practice; for in the latter he had had but little experience. He then stated the ground of his theory in the action of that anæsthetic, in its chemical influence upon the circulatory and respiratory system.

"Dr. Clark said the question is: Is it most serviceable for lying-in women to take chloroform or not to take it? He alluded to the success of Dr. Burwell's one hundred and eighty cases, of as many more of Dr. Barker, and of the success of other physicians. He thought where their experience confirmed so much that is at variance with Dr. Spencer's theory, that gentleman might question the correctness of that theory, He thought the rules for its administration had been so definitely laid down by Dr. Burwell, that an intelligent man would not be likely to err.

"Dr. Shipman said he had used chloroform in a good many hundred cases for surgical operations, and without any serious result. He considered it a safe remedy. He had used it in only eight cases of midwifery. It did not, in any of these cases, prolong labor. He had administered it repeatedly to the inferior animals—birds, rats, mice, and turtles. He thought it more destructive to them than to man.

"Dr. Burwell stated that he thought the danger consisted in the manner in which it was administered. If given in large quantities at first, it was dangerous. It should be inhaled in small quantities. The patient should be allowed to inhale the air with it.

"Dr. Cock said it was quite too late to question its utility; and related some of the cases which had come under his observation, where most decided benefit attended its use."

We have had some experience with chloroform in midwifery. Given with proper care we conceive it is unattended by much, if any risk of accident. We have generally, however, preferred that the responsibility of its use should rest with the patient, or friends. We are satisfied that it sometimes retards labor, but this objection is more than compensated for by the comfort it affords, and the refreshing influence derived from it when not carried to the point of insensibility. We do not hesitate to employ it, and are always glad when patients or friends solicit it.

### *Visitation of Medical Colleges.*

"Dr. Rockwell made a verbal report in behalf of the censors of the Southern District. In accordance with a resolution passed at the last session, they had attended some of the examinations of the medical colleges in the city of New York. The committee found it impossible to attend all the examinations, which continue through nearly two weeks. He was pleased to say that those students whom he had marked as unfit to receive diplomas were rejected. So far as the committee had opportunity to observe, the examinations were honerably conducted."

*Increasing number of Delegates to the Society.*

"Dr. Blatchford read a paper on the propriety of increasing the number of delegates to this Society, making a ratio of one to every ten from the county societies, and each delegate to hold his office for two years, and offered a resolution that a committee be appointed to confer with a committee of the Legislature for an amendment of the statute law on this subject.

"Dr. Clark said it was an important matter to alter the fundamental organization of the Society. The changes proposed would entitle New York to ninety members, which is almost equal to this Society's present members.

"Dr. Ely suggested that it would be well for each county society to be entitled to delegates corresponding to the number of members of Assembly from such county; and that the resolution should be so amended.

"Dr. Wood said, this body was intended for an executive body—to it the people and the Legislature look for a healthful medical influence; and that this Society should carefully examine the condition of subordinate societies before a larger representation is admitted from them.

"Dr. Blatchford withdrew his resolution, when the subject was referred to a committee consisting of Drs. Coventry, Rockwell, Cock, Blatchford and Thompson."

Subsequently Dr. Coventry, chairman of committee, reported as follows:

"*Resolved*, That a special committee of three members, residing in this city, be appointed to confer with the medical gentlemen of the legislature, as to the propriety and practicability of the passage at the present session of the legislature, of an act having in view the more equal representation of the medical talent of the state in this society."

*Delegates to the American Medical Association.*

"Leave was granted to Dr. Bradford to substitute the following preamble and resolutions for the paper offered by him yesterday:

"Whereas, it is essential to the interests of the whole medical profession of the United States, that the system of representation by delegates in the American Medical Association, should be established upon a free, equal, and liberal basis, so as to secure the attendance of a large number of respectable physicians from every state in the Union; and

"Whereas, the percentage of representation, as established by the present constitution of the association, is deemed to be liberal and just, in so far as it applies to State, County, and Voluntary Medical Societies; therefore

"*Resolved*, That the Medical Society of the State of New York deprecates, and considers unadvisable, any change in the constitution of the association, which would diminish their number, or exclude delegates from State, County, and Voluntary Medical Associations.

"*Resolved*, That the Medical Society of the State of New York approves of the system of representation to the association, embraced in the amendments proposed and adopted at the last meeting of the American Medical Association, and hopes that the said amendments may be favorably received by the next meeting, and be adopted.

"*Resolved*, That a certified copy of the above preamble and resolutions be transmitted by the secretary to Beverley R. Wellford, M. D., president of the American Medical Association, with the request that it will present the same to the association at its next meeting.

"The preamble and resolutions were adopted."

*Petition for a Room in the Capitol.*

"Dr. Van Buren offered a resolution to appoint a committee to petition the legislature for a room in the capitol for the exclusive use of the Medical Society. The resolution was adopted."

We see no reference, in the proceedings, to a semi-annual meeting of the society. This plan is, therefore, we infer, abandoned.

